# No association between three polymorphisms (rs1800629, rs361525 and rs1799724) in the tumor necrosis factor-α gene and susceptibility to prostate cancer: a comprehensive meta-analysis

**DOI:** 10.1186/s41065-020-00125-1

**Published:** 2020-04-07

**Authors:** Lei Yin, Chuang Yue, Hongwei Jing, Hongyuan Yu, Li Zuo, Tao Liu

**Affiliations:** 1 Department of Urology, The First Affiliated Hospital of China Medical University, Shenyang, 110001 P.R. China; 2 Department of Urology, Changzhou No. 2 People’s Hospital Affiliated to Nanjing Medical University, Changzhou, 213003 Jiangsu Province China

**Keywords:** Tumor necrosis factor-alpha, Prostate cancer, Polymorphism, Meta-analysis, Susceptibility

## Abstract

**Background:**

Inflammation is one of the factors associated with prostate cancer. The cytokine tumor necrosis factor-alpha (TNF-α) plays an important role in inflammation. Several studies have focused on the association between TNF-α polymorphisms and prostate cancer development. Our meta-analysis aimed to estimate the association between TNF-α rs1800629 (− 308 G/A), rs361525 (− 238 G/A) and rs1799724 polymorphisms and prostate cancer risk.

**Methods:**

Eligible studies were identified from electronic databases (PubMed, Embase, Wanfang and CNKI) using keywords: TNF-α, polymorphism, prostate cancer, until Nov 15, 2019. Odds ratios (ORs) with 95% confidence intervals (CIs) were applied to determine the association from a quantitative point-of-view. Publication bias and sensitivity analysis were also applied to evaluate the power of current study. All statistical analyses were done with Stata 11.0 software.

**Results:**

Twenty-two different articles were included (22 studies about rs1800629; 8 studies for rs361525 and 5 studies related to rs1799724). Overall, no significant association was found between rs1800629 and rs1799724 polymorphisms and the risk of prostate cancer in the whole (such as: OR = 1.03, 95% CI = 0.92–1.16, *P* = 0.580 in the allele for rs1800629; OR = 0.95, 95% CI = 0.84–1.07, *P* = 0.381 in the allele for rs1799724). The rs361525 polymorphism also had no association with prostate cancer in the cases (OR = 0.93, 95% CI = 0.66–1.32, *P* = 0.684 in the allele) and ethnicity subgroup. The stratified subgroup of genotype method, however, revealed that the rs361525 variant significantly decreased the risk of prostate cancer in the Others (OR = 0.65, 95% CI = 0.47–0.89, *P* = 0.008, A-allele vs G-allele) and PCR-RFLP (OR = 2.68, 95% CI = 1.00–7.20, *P* = 0.050, AG vs GG or AA+AG vs GG) methods.

**Conclusions:**

In summary, the findings of the current meta-analysis indicate that the TNF-α rs1800629, rs361525 and rs1799724 polymorphisms are not correlated with prostate cancer development, although there were some pooled positive results. Further well-designed studies are necessary to form more precise conclusions.

## Background

Prostate cancer (PCA) is the second most frequent tumor in men worldwide, with 1.27 million new cases and 0.35 million deaths in 2018 [1, 2]. The incidence and mortality of PCA are correlated with increasing age, and the average age at the time of diagnosis is over 66 years in some regions. Additionally, there is also evidence of an association between ethnicity and PCA; for example, the incidence rate in African-American men is 158.3 newly diagnosed cases/100,000, which is higher than that in White men, and their mortality is about twice that of White men according to Panigrahi et al. [3]. Several factors may contribute to this disparity, such as differences in diet, habits/customs, and genetic/environmental factors.

There is growing evidence that chronic inflammation is involved in the regulation of cellular events in prostate carcinogenesis, including disruption of the immune response and regulation of the tumor microenvironment [4]. One of the best surrogates of chronic inflammation in PCA is the cytokine tumor necrosis factor alpha (TNF-α) [5, 6]. Chadha et al. indicated the median TNF-α levels in serum was significantly higher (*P* < 0.05) in the control group (5.12 pg/ml) than in the localized PCA group (2.20 pg/ml). Moreover, TNF-α was the strongest single predictor between localized and metastatic PCA (Area Under Curve, AUC = 0.992) and was higher than the PSA value (AUC = 0.963). Taken together, these results suggest that TNF-α may be considered a novel serum biomarker for the diagnosis of PCA [7].

The TNF-α gene, also termed DIF/TNFSF2/TNLG1F, is located in the class III region of the major histocompatibility complex (MHC III) and mapped to chromosome 6p21.33 with 4 exons [8, 9]. Several single nucleotide polymorphisms (SNPs) in this gene have been widely reported and have been associated with the risk of several cancers, such as PCA, breast cancer, and lung cancer [10–12]. Rs1800629 is one of the most common SNPs, with a G to A transition at the − 308 nucleotide in the promoter of the transcription initiation site, which may affect the serum expression of TNF-α [13]. Another common SNP named rs361525 is located at the − 238 site, where a G to A substitution is shown, and may influence TNF-α in the serum [14]. The rs1799724 (C to T transition) and rs1799964 (T to C transition) SNPs have been reported in recent years [15, 16]; however, to date, it is not known whether these two SNPs can affect the expression of TNF-α.

Previously, two meta-analyses focused on TNF-α polymorphisms and PCA risk have been published: Cai et al. identified 12 case-control studies and concluded that the rs1800629 polymorphism had an increased association with PCA risk in the GA vs. GG genetic model (OR = 1.19, 95% CI = 1.04–1.37) [17]. Ma et al., however, suggested that the rs361525 polymorphism was not associated with PCA, and the rs1800629 polymorphism, which is also the susceptible SNP for PCA, only had a significant association in healthy volunteers (AG vs. GG: OR = 1.47, 95% CI = 1.04–2.08) [18]. Due to these inconclusive results, as well as the publication of some additional studies, it was necessary to re-combine all of the articles, including 22 different case-control studies [15, 16, 19–36], to conduct an updated meta-analysis.

## Methods

### Literature search and inclusion criteria

We performed a literature search for all eligible articles regarding the association between four TNF-α polymorphisms and PCA risk on multiple electronic databases, including PubMed, Embase, Wanfang and CNKI, using the following keywords: ‘tumor necrosis factor alpha OR TNF-α’ AND ‘polymorphism OR variation OR mutation’ AND ‘prostate cancer OR carcinoma OR neoplasm OR tumor’ until Nov 15, 2019.

Relevant studies were selected based on the following inclusion criteria: (1) case-control studies addressing the correlation between a TNF-α polymorphism and PCA risk; (2) studies containing sufficient genotype data on both the cases and controls; and (3) the largest sample sizes were selected among articles with overlapping study groups. The exclusion criteria were (1) conference abstracts, case reports, reviews and duplicated information; and (2) inadequate genotype data.

### Data extraction

The following data were gathered from each eligible study: the first author’s name, publication year, country, sample size for the case and control groups, source of control, Hardy-Weinberg equilibrium (HWE) of the controls, genotyping techniques and the genotype of the cases and controls.

### Statistical analysis

The strength of the association between the four TNF-α polymorphisms and PCA susceptibility was measured by the odds ratio (OR) with 95% confidence interval (CI) in 3 (allele, heterozygous and dominant) genetic models. The significance of the pooled OR was assessed by the *Z*-test, and *P* < 0.05 was considered to be statistically significant. The between-study heterogeneity was evaluated by the *Q*-test. In cases where significant heterogeneity was detected, if *P* < 0.1, indicating the presence of heterogeneity, a random-effects model was selected; otherwise, a fixed-effects model was applied [37, 38]. Publication bias was inspected using Begg’s test, and Egger’s test was used to measure the degree of asymmetry. In both tests, *P* < 0.05 was considered statistically significant [39]. The HWE of the control group was specified through the chi-square test, where *P* < 0.05 was considered significant [40]. Sensitivity analyses were done to evaluate whether a single study influenced the overall pooled results by omitting each study in turn. All statistical tests used in this study were performed using Stata (version 11.0; StataCorp LP, College Station, TX).

## Results

### Characteristics of selected studies

**Fig. 1:**
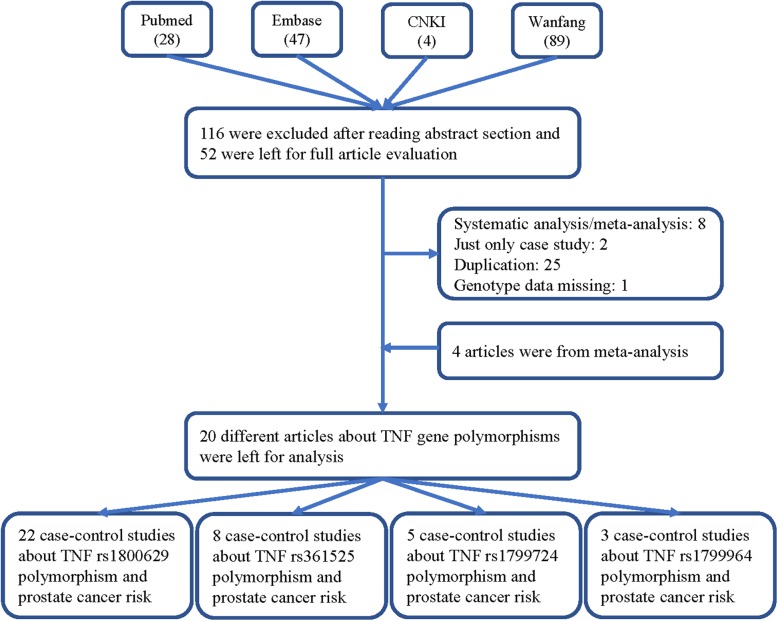
A flowchart illustrating the search strategy about TNF-α rs1800629, rs361525, rs1799724 and rs1799964 polymorphisms and PCA risk was shown

**Fig. 2:**
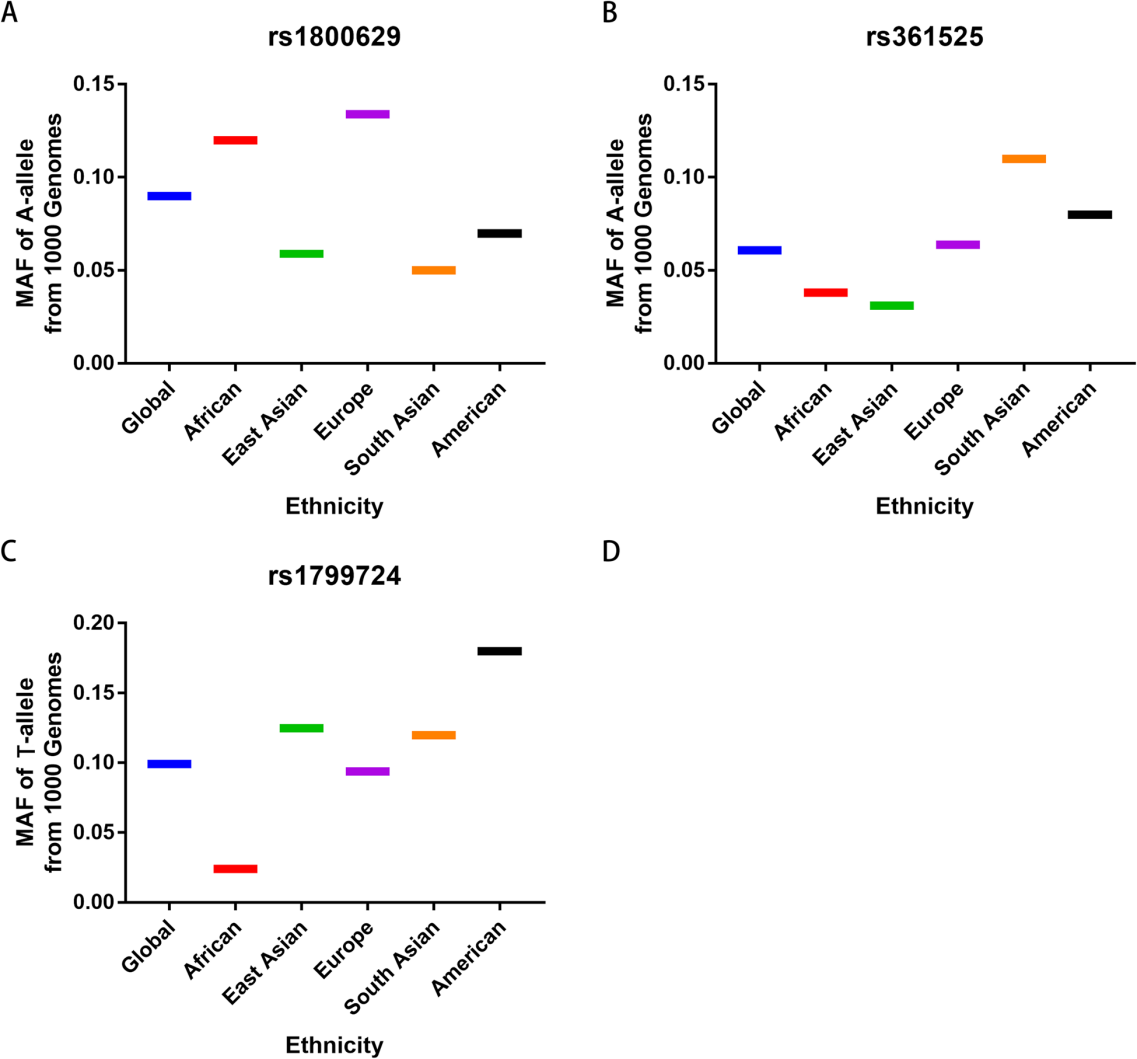
MAF for the TNF-α rs1800629, rs361525 and rs1799724 polymorphsms from 1000 Genomes Browser. Vertical line, MAF; Horizontal line, ethnicity type. EAS: East Asian; EUR: European; AFR: African; AMR: American; SAS: South Asian

A total of 168 published articles were retrieved from the PubMed, Embase, Wanfang and CNKI databases in accordance with the selection criteria. Finally, 20 different articles (22 case-control studies) were included in our meta-analysis (Table 1, Fig. 1) [15, 16, 19–36]. Of the 22 studies, TNF-α rs1800629 was analyzed in 22 studies; rs361525, in 8 studies; rs1799724, in 5 studies; and rs1799964, in 3 studies. Only three available reports investigated rs1799964 and PCA susceptibility, so we did not analyze this association. Table 1 shows the features and related information of the included studies. In addition, we checked the Minor Allele Frequency (MAF) reported for the five main worldwide populations in the 1000 Genomes Browser for each SNP: East Asian (EAS), European (EUR), African (AFR), American (AMR), and South Asian (SAS) (Fig. 2).

**Table 1:** Characteristics of the studies eligible for current meta-analysis

Author	Year	Country	Ethnicity	Case	Control	SOC	Cases			Controls			HWE	Genotype
							MM	MW	WW	MM	MW	WW		
rs1800629														
Jones	2013	USA	African-American	279	535	HB	5	103	171	14	153	368	0.687	Illumina’s Golden gate
Zabaleta	2008	USA	African-American	67	130	HB	2	9	56	3	33	94	0.958	Sequence
Berhane	2012	India	Asian	150	150	HB	6	24	120	1	18	131	0.662	ARMS-PCR
Wu	2003	China-Taiwan	Asian	96	126	HB	2	20	74	1	22	103	0.882	PCR-RFLP
Alidoost	2019	Iran	Asian	100	110	HB	0	16	84	0	14	96	0.476	PCR-RFLP/ARMS-PCR
Kesarwani	2009	India	Asian	197	256	HB	1	21	175	4	37	215	0.115	PCR-RFLP
Ali	2019	Iraq	Asian	30	30	PB	12	18	0	24	6	0	0.543	PCR-RFLP
Ge	2007	China	Asian	245	245	HB	2	39	204	2	48	195	0.609	TaqMan
Dluzniewski	2012	USA	Caucasian	468	468	HB	14	113	341	6	126	336	0.125	MassArray
Pardo	2019	Venezuela	Caucasian	40	40	HB	0	6	34	0	11	29	0.313	PCR-RFLP
Zabaleta	2008	USA	Caucasian	479	400	HB	9	148	322	10	118	272	0.505	Sequence
Sáenz-López	2008	Spain	Caucasian	296	310	PB	5	70	221	2	52	256	0.714	TaqMan
Moore	2009	USA	Caucasian	949	857	PB	21	228	700	11	205	641	0.231	TaqMan
Danforth	2008	USA	Caucasian	1155	1380	PB	26	336	793	45	418	926	0.795	TaqMan/MGBEclipse assay
Danforth	2008	USA	Caucasian	1111	1125	PB	25	294	792	33	286	806	0.217	TaqMan/MGBEclipse assay
Ribeiro	2012	Portugal	Caucasian	449	557	PB	8	115	326	7	143	407	0.155	TaqMan
Wang	2009	USA	Caucasian	251	250	PB	12	79	160	9	69	172	0.529	TaqMan
Bandil	2017	India	Asian	105	115	HB	9	15	81	4	7	104	< 0.001	ARMS-PCR
Omrani	2008	Iran	Asian	41	105	HB	0	36	5	3	99	3	< 0.001	ASO-PCR
McCarron	2002	United Kingdom	Caucasian	239	220	HB	6	66	167	13	57	150	0.023	ARMS-PCR
OH	2000	USA	Caucasian	73	73	HB	0	53	20	0	53	20	< 0.001	allele-specific PCR
Zhang	2010	USA	Caucasian	116	128	PB			116			128		CBMALD-TOF-MS
rs361525														
Pardo	2019	Venezuela	Caucasian	40	40	HB	0	4	36	0	1	39	0.936	PCR-RFLP
OH	2000	USA	Caucasian	73	73	HB	0	23	50	0	23	50	0.11	allele-specific PCR
Zabaleta	2008	USA	Caucasian	471	385	HB	6	41	424	0	39	346	0.295	Sequence
Alidoost	2019	Iran	Asian	100	110	HB	0	10	90	0	5	105	0.807	PCR-RFLP/ARMS-PCR
Danforth	2008	USA	Caucasian	1114	1126	PB	1	121	992	3	100	1023	0.737	TaqMan/MGBEclipse assay
Ge	2007	China	Asian	245	245	HB	0	10	235	0	22	223	0.461	TaqMan
Zabaleta	2008	USA	African-American	64	126	HB	0	6	58	2	10	114	0.006	Sequence
Bandil	2017	India	Asian	105	115	HB	12	60	33	20	86	9	< 0.001	ARMS-PCR
rs1799724														
Danforth	2008	USA	Caucasian	1139	1378	PB	13	203	923	14	254	1110	0.9	TaqMan/MGBEclipse assay
Danforth	2008	USA	Caucasian	1108	1101	PB	17	183	908	19	220	862	0.257	TaqMan/MGBEclipse assay
Kesarwani	2009	India	Asian	197	256	HB	4	57	136	4	56	196	1	PCR-RFLP
Zabaleta	2008	USA	African-American	464	372	HB	6	59	399	8	41	323	< 0.001	Sequence
Zabaleta	2008	USA	Caucasian	6	14	HB	3	0	3	7	0	7	< 0.001	Sequence
rs1799964														
Danforth	2008	USA	Caucasian	1142	1375	PB	60	361	721	58	441	876	0.791	TaqMan/MGBEclipse assay
Danforth	2008	USA	Caucasian	1143	1155	PB	54	370	719	64	377	714	0.129	TaqMan/MGBEclipse assay
Kesarwani	2009	India	Asian	197	256	HB	90	64	43	83	91	82	< 0.001	PCR-RFLP

*HB* hospital-based, *PB* population-based, *SOC* source of control, *PCR-FLIP* polymerase chain reaction and restrictive fragment length polymorphism; *ARMS* amplification refractory mutation system, *HWE* Hardy–Weinberg equilibrium of control group, *W* wild type-allele, *M* mutant-allele

### Pooled analysis results

**Fig. 3:**
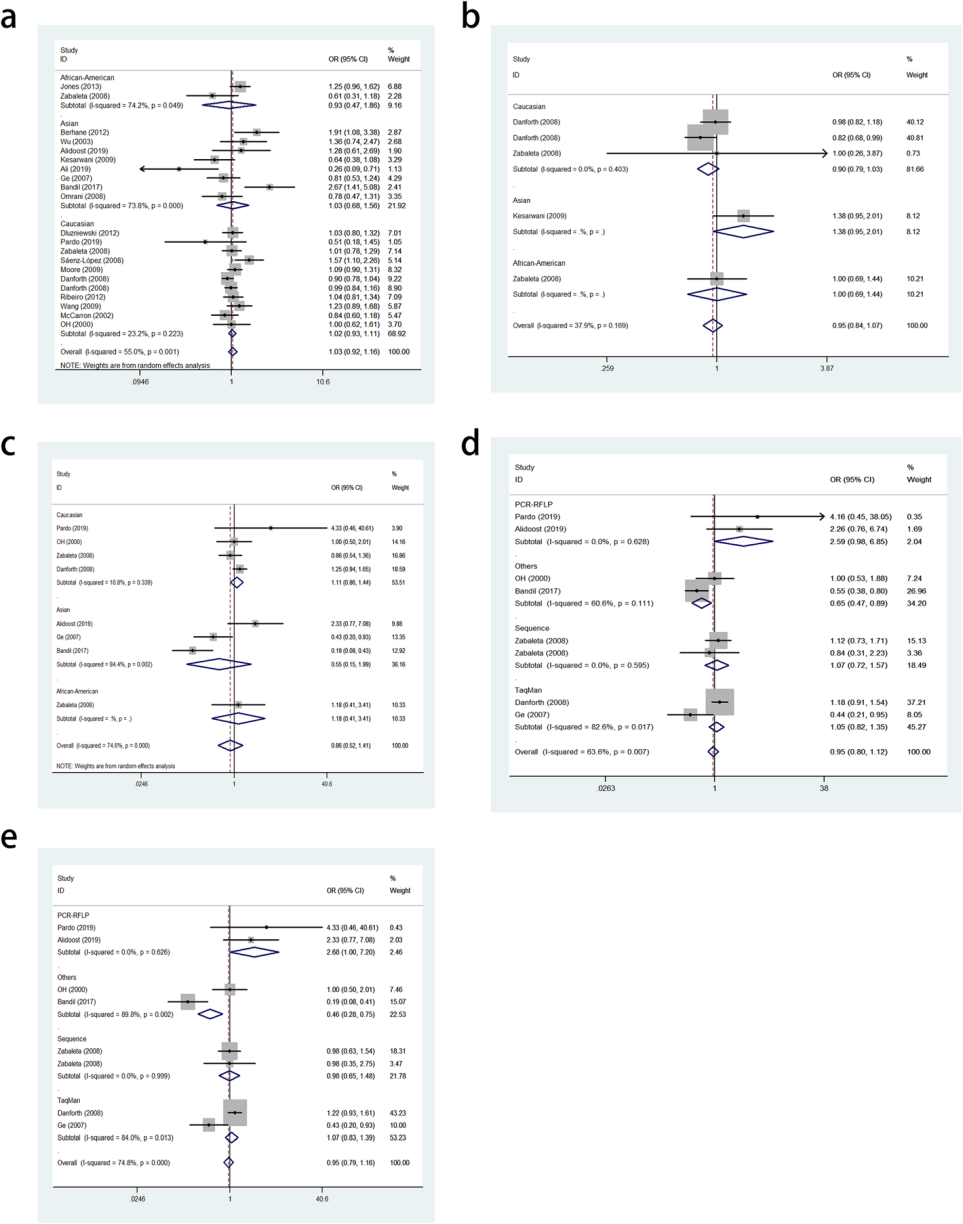
Meta-analysis. **a**. Forest plots of TNF-α rs1800629 polymorphism and PCA risk (A-allele vs. G-allele). **b**. Forest plot of TNF-α rs1799724 polymorphism and PCA risk (T-allele vs. C-allele). **c**. Forest plot of TNF-α rs361525 polymorphism and PCA risk (AA vs. GG). **d**. Forest plot of TNF-α rs361525 polymorphism and PCA risk (A-allele vs. G-allele) on subgroup of genotyping method (Others). **e**. Forest plot of TNF-α rs361525 polymorphism and PCA risk (A-allele vs. G-allele) on subgroup of genotyping method (PCR-RFLP)

Overall, the findings did not support an association between the TNF-α rs1800629 polymorphism and PCA susceptibility in the allele (OR = 1.03, 95% CI = 0.92–1.16, *P* = 0.580, Fig. 3a), heterozygous (OR = 1.04, 95% CI = 0.93–1.17, *P* = 0.486) and dominant (OR = 1.06, 95% CI = 0.94–1.18, *P* = 0.353) genetic models. To evaluate the power and stability, some studies not consistent with HWE were excluded, and similar results were obtained. Stratified analyses by ethnicity, source of control and genotyping methods were conducted, and no significant association was detected (Table 3).

For the TNF-α rs1799724 polymorphisms, no significant associations were identified in the cases and subgroups. Further, the rs1799724 polymorphism was not significantly associated with PCA in the allele (OR = 0.95, 95% CI = 0.84–1.07, *P* = 0.381, Fig. 3b), heterozygous (OR = 1.01, 95% CI = 0.80–1.27, *P* = 0.951) and dominant genetic models (OR = 0.95, 95% CI = 0.83–1.07, *P* = 0.390).

For the TNF-α rs361525 polymorphism, although no association was found in the allele (OR = 0.93, 95% CI = 0.66–1.32, *P* = 0.684), heterozygous (OR = 0.86, 95% CI = 0.52–1.41, *P* = 0.542, Fig. 3c) and dominant models (OR = 0.85, 95% CI = 0.52–1.39, *P* = 0.525), for HWE, ethnicity and source of control, pooled significant relationships were observed in genotyping subgroups, such as Others (OR = 0.65, 95% CI = 0.47–0.89, *P* = 0.008 for A-allele vs. G-allele, Fig. 3d) and PCR-RFLP (OR = 2.68, 95% CI = 1.00–7.20, *P* = 0.050, Fig. 3e).

### Heterogen

#### Heterogeneity and publication bias

As shown in Table 2, heterogeneity among the studies was found in all three genetic comparisons for all 3 SNPs (rs1800629, rs361525 and rs1799724).

**Table 2:** The pooled ORs and 95%CIs for the association between TNF polymorphisms and prostate cancer susceptibility in total and stratified analysis

Variables	N	Case/Control	M-allele vs. W-allele OR(95%CI) *P*_h_*P*	MW vs. WW OR(95%CI) *P*_h_*P*	MM + MW vs. WW OR(95%CI) *P*_h_*P*
rs1800629					
Total	22	6936/7619	1.03 (0.92–1.16)0.001 0.580	1.04 (0.93–1.17)0.040 0.486	1.06 (0.94–1.18)0.013 0.353
HWE	18	7485/6792	1.03 (0,92–1.16)0.006 0.584	1.04 (0,93–1.16)0.091 0.509	1.05 (0,94–1.17)0.051 0.429
Ethnicity					
Asian	8	964/1137	1.03 (0.68–1.56)0.000 0.881	1.04 (0.70–1.56)0.038 0.845	1.09 (0.70–1.71)0.006 0.698
Caucasian	12	5626/5817	1.01 (0.94–1.08)0.223 0.838	1.02 (0.94–1.11)0.525 0.672	1.02 (0.94–1.11)0.433 0.625
African-American	2	346/665	0.93 (0.47–1.86)0.049 0.843	0.87 (0.28–2.67)0.009 0.804	0.90 (0.34–2.37)0.016 0.829
SOC					
HB	14	2579/2973	1.02 (0.86–1.22)0.012 0.787	1.00 (0.81–1.22)0.023 0.972	1.01 (0.82–1.24)0.012 0.787
PB	8	4357/4646	1.04 (0.89–1.22)0.009 0.600	1.04 (0.94–1.14)0.298 0.483	1.04 (0.95–1.14)0.199 0.425
Genotyping					
Others	5	977/1309	1.07 (0.91–1.26)0.420 0.420	0.97 (0.62–1.53)0.021 0.900	1.07 (0.79–1.45)0.079 0.668
Sequencing	2	546/530	0.94 (0.75–1.19)0.166 0.608	0.76 (0.34–1.70)0.055 0.505	0.80 (0.41–1.55)0.086 0.506
TaqMan	7	4456/4733	1.04 (0.92–1.17)0.081 0.520	1.02 (0.93–1.12)0.278 0.638	1.02 (0.93–1.12)0.152 0.672
PCR-RFLP	5	463/562	0.74 (0.43–1.28)0.030 0.280	0.90 (0.63–1.29)0.263 0.565	0.89 (0.63–1.26)0.186 0.520
ARMS-PCR	3	494/485	1.56 (0.74–3.29)0.001 0.239	1.28 (0.93–1.78)0.163 0.135	1.54 (0.80–2.97)0.024 0.192
rs361525					
Total	8	2212/2222	0.93 (0.66–1.32)0.007 0.684	0.86 (0.52–1.41)0.000 0.542	0.85 (0.52–1.39)0.000 0.525
HWE	6	2043/1979	1.11 (0,91–1.35)0.111 0.321	1.02 (0,69–1.52)0.055 0.905	1.05 (0,73–1.52)0.803 0.794
Ethnicity					
Asian	3	450/470	0.72 (0.34–1.50)0.039 0.380	0.55 (0.15–1.99)0.002 0.360	0.54 (0.15–2.00)0.001 0.357
Caucasian	4	1698/1624	1.16 (0.94–1.44)0.673 0.164	1.16 (0.94–1.44)0.673 0.164	1.16 (0.94–1.44)0.673 0.164
African-American	1	64/126	–	–	–
Genotyping					
Others	2	178/188	0.65 (0.47–0.89)0.111 0.008	0.44 (0.09–2.25)0.002 0.326	0.44 (0.08–2.28)0.002 0.325
Sequencing	2	535/511	1.07 (0.72–1.57)0.595 0.746	0.90 (0.59–1.38)0.590 0.633	0.98 (0.65–1.48)0.999 0.936
PCR-RFLP	2	140/150	2.59 (0.98–6.85)0.628 0.055	2.68 (1.00–7.20)0.626 0.050	2.68 (1.00–7.20)0.626 0.050
TaqMan	2	1359/1371	0.77 (0.30–2.01)0.017 0.599	0.78 (0.28–2.20)0.011 0.640	0.77 (0.28–2.13)0.013 0.620
rs1799724					
Total	5	2914/3121	0.95 (0.84–1.07)0.169 0.381	1.01 (0.80–1.27)0.054 0.951	0.95 (0.83–1.07)0.120 0.390
HWE	3	2444/2735	0.99 (0,78–1.26)0.042 0.930	0.98 (0,74–1.30)0.037 0.896	0.99 (0,75–1.30)0.032 0.931
Caucasian	3	2253/2493	0.90 (0.79–1.03)0.403 0.115	0.88 (0.76–1.02)0.196 0.082	0.88 (0.76–1.02)0.400 0.089

*P*_h_: value of *Q*-test for heterogeneity test; *P*: *Z*-test for the statistical significance of the OR; *HB* hospital-based, *PB* population-based, *SOC* source of control, *PCR-FLIP* polymerase chain reaction and restrictive fragment length polymorphism, *ARMS* amplification refractory mutation system *HWE*, Hardy–Weinberg equilibrium of control group, *W* wild type-allele, *M* mutant-allele

**Fig. 4:**
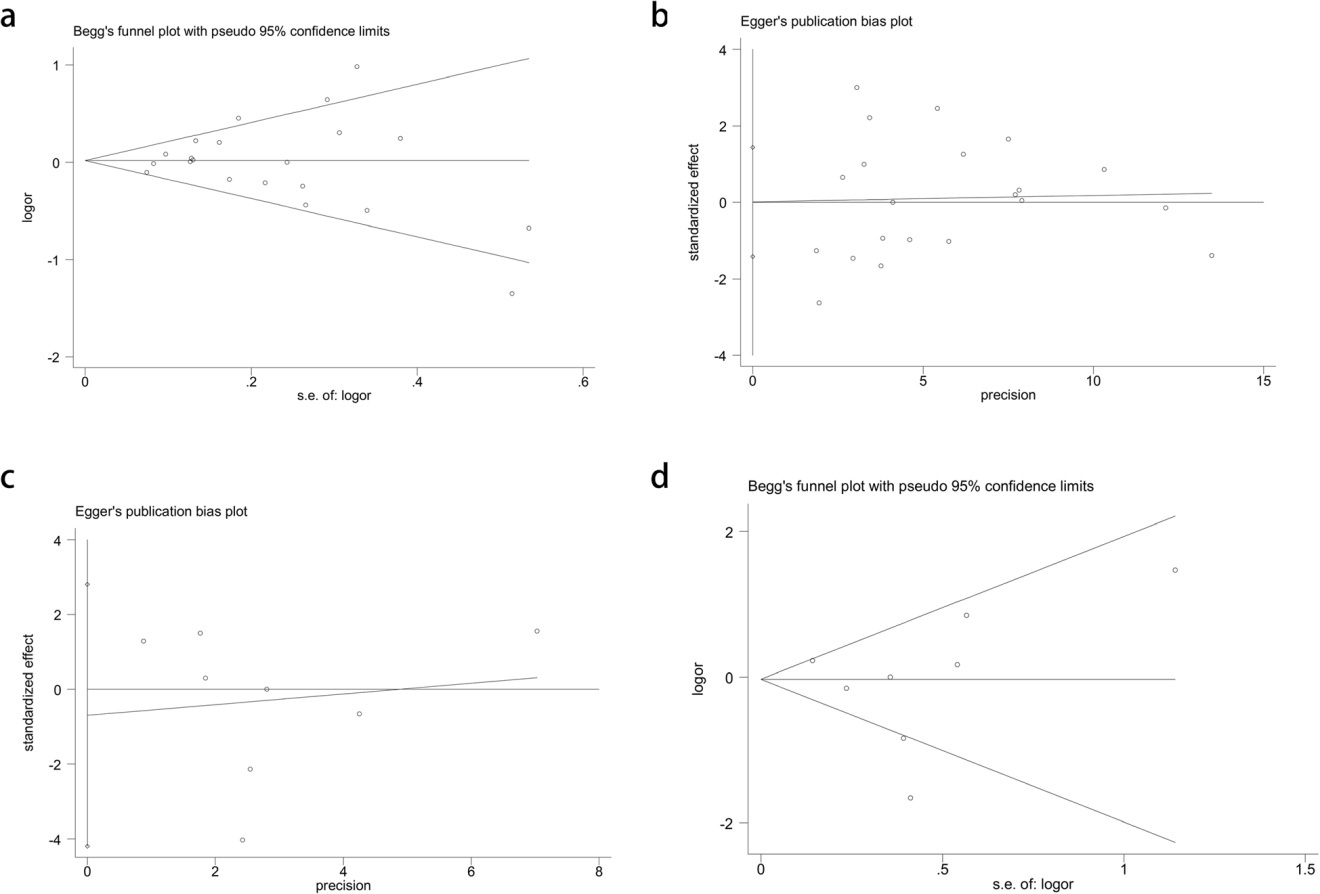
Publication bias. **a**. Begg’s funnel plot for publication bias test (A-allele vs. G-allele). **b**. Egger’s publication bias plot (A-allele vs. G-allele). **c**. Begg’s funnel plot for publication bias test (A-allele vs. G-allele). **d**. Egger’s publication bias plot (A-allele vs. G-allele)

The publication bias was assessed by applying Begg’s funnel plot and Egger’s test. Based on the samples and publications, we tested two SNPs, rs1800629 and rs361525. The shape of the funnel plots was symmetrical, and the Egger’s test supported no existence of publication bias in any of the three comparisons for the rs1800629 (*t* = 0.01, *p* = 0.989 for Egger’s test; *z* = 0.21, *p* = 0.833 for Begg’s test, Fig. 4a, b) and rs361525 (*t* = − 0.3, *p* = 0.765 for Egger’s test; z = − 0.12, *p* = 1 for Begg’s test, Fig. 4c, d) polymorphisms (Table 3).

**Table 3:** Publication bias tests (Begg’s funnel plot and Egger’s test for publication bias test) for rs1800629 and rs361525 polymorphisms

Egger’s test						Begg’s test	
Genetic type	Coefficient	Standard error	*t*	*P* value	95%CI of intercept	*z*	*P* value
rs1800629							
A-allele vs. G-allele	0.009	0.681	0.01	0.989	(−1.418–1.437)	0.21	0.833
AG vs. GG	0.331	0.528	0.63	0.539	(−0.779–1.440)	0.1	0.922
AA+AG vs. GG	0.046	0.619	0.07	0.941	(−1.249–1.341)	0.33	0.74
rs361525							
A-allele vs. G-allele	−0.216	1.259	0.17	0.87	(−2.866–3.297)	0.12	0.902
AG vs. GG	−0.293	0.935	−0.3	0.765	(−2.582–1.996)	− 0.12	1
AA+AG vs. GG	−0.303	0.938	−0.3	0.757	(−2.599–1.991)	− 0.12	1

### Sensitivity analysis

**Fig. 5:**
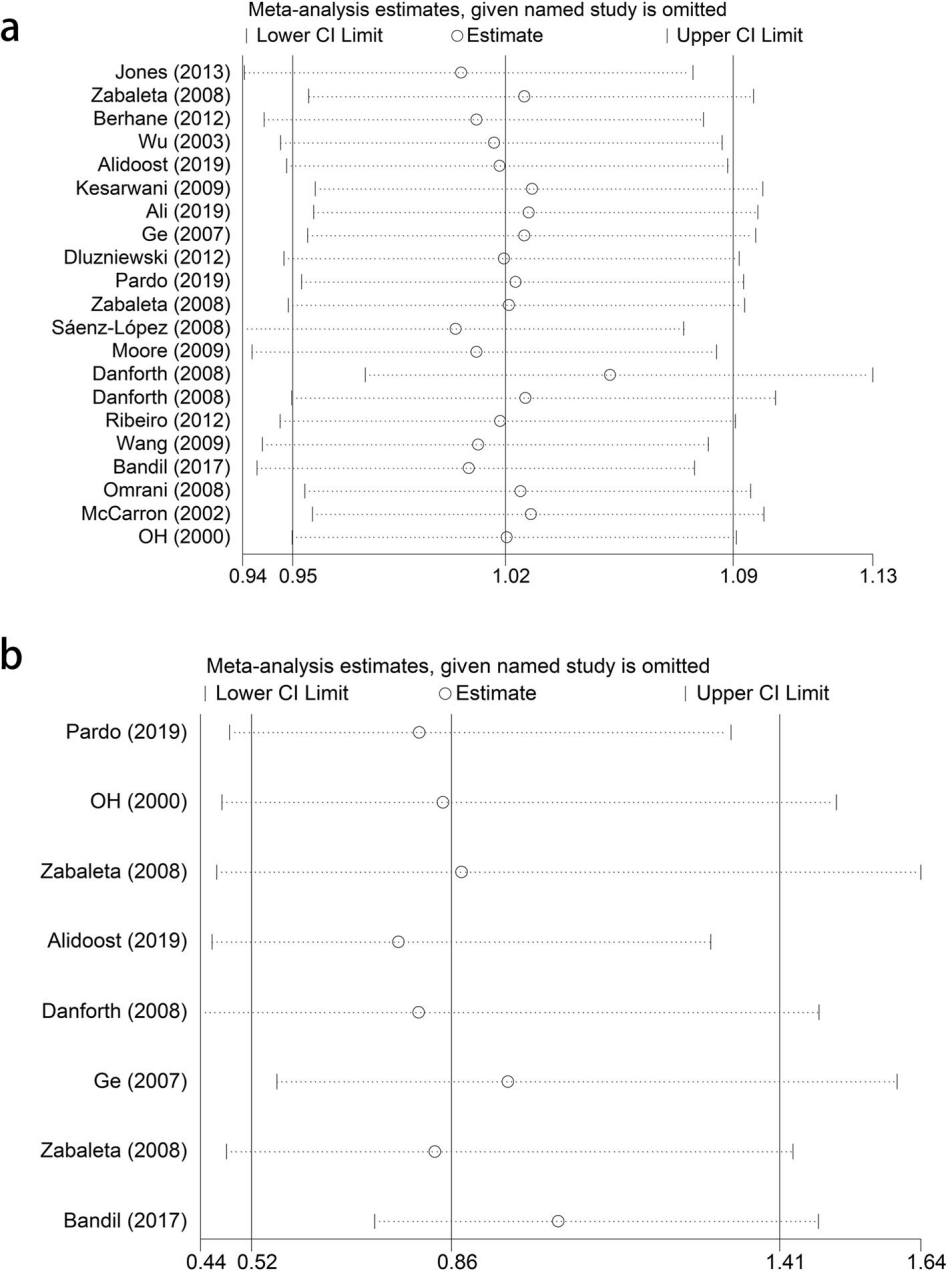
Sensitivity analysis. **a**. Sensitivity analysis for TNF-α rs1800629 polymorphism and RA risk (A-allele vs. G-allele). **b**. Sensitivity analysis between TNF-α rs361525 polymorphism and RA risk (A-allele vs. G-allele)

We performed sensitivity analyses to assess the effect of a specific publication on the overall estimate. Similar with publication bias, we also analyzed both rs1800629 and rs361525 (Fig. 5a, b), and no significant changes were observed when excluding each study in any of the three genetic models (allele, heterozygous and dominant). Thus, the final pooled results are both stable and reliable.

## Discussion

There is evidence to suggest that chronic inflammation is prevalent in the adult prostate and may contribute to disease development in the form of promoting tumor initiation and progression [5, 41]. Therefore, chronic inflammation has been considered an enabling characteristic in the development of cancers [42], such as PCA. Several previous epidemiological studies have been explored to make a connection between inflammation and PCA development, showing evidence that associates symptomatic prostatitis with PCA risk [43–45]. For example, men with prostatitis have increased serum PSA levels, and while a medical diagnosis for prostatitis symptoms may be received initially, they may be screened for PCA and might be diagnosed with PCA in the end. Furthermore, many men with prostatic inflammation without symptoms also have increased PSA values, which may increase the odds of visits to the doctor, and they may be identified as having PCA [45]. Taken together, these observations indicate that the detection of inflammation in the prostate may be helpful for us to better identify PCA patients; however, there are no specific biomarkers of prostate inflammation to date.

Studying both pro- and anti-inflammatory cytokine genes is essential for PCA [21]. TNF-α, as a main mediator of inflammation, has a vital role in PCA development [10]. By considering the capacity of TNF-α promoter SNPs (rs1800629 and rs361525), and the influence of their gene expression [13, 14], these two SNPs have been identified as potential functional variants and as novel biomarkers for the early detection for PCA susceptibility.

Several studies and two meta-analyses have examined the association between TNF-α gene polymorphisms and PCA risk [15–36]. Nevertheless, the findings were inconsistent, possibly due to the small samples or relatively low statistical power of the included studies. Therefore, a current, updated meta-analysis with a comprehensive assessment that included more eligible studies was performed to evaluate the impact of TNF-α gene polymorphisms (rs1800629, rs361525 and rs1799724) on PCA susceptibility, which may overcome the aforementioned disadvantages [15, 16, 19–36]. For the TNF-α rs1800629 polymorphism, the findings from 20 studies, including 6936 cases and 7619 controls, did not support an association between this variant and PCA risk [15, 16, 19–36]. To the best of our knowledge, for the rs1799724 [15, 16, 35] polymorphism, which was analyzed for the first time, no significant association was detected from 3 studies, which included 2914 cases and 3121 controls. For the rs361525 polymorphism [15, 20, 21, 24, 28, 30, 35], pooled significant relationships were observed in the genotyping method subgroups. Cumulatively, we believe no association exists between the four common TNF-α polymorphisms and PCA risk based on the current evidence.

Despite a comprehensive analysis of the current associations between the four TNF-α polymorphisms and the risk of developing PCA, there are some limitations that should be considered. First, the number of samples remains insufficient, especially for the rs1799724 and rs1799964 polymorphisms and ethnicities in some polymorphisms, such as African-American, Asian, African and mixed populations, which perhaps leads to imbalance and publication bias. Second, gene-gene, SNP-SNP and gene-environment interactions should be taken into consideration. Other covariates, including prostate health index, age, family history, environmental factors, Gleason score, TNM stage and living habits, should be better observed, which will help us to draw an exact conclusion. Third, the protein expression level of TNF-α in different polymorphisms should also be observed and be reevaluated by meta-analysis in the future research.

In summary, our study presents evidence that three of the most common TNF-α polymorphisms (rs1800629, rs361525 and rs1799724) are not associated with PCA risk, which should be verified in the future, but they may be poised to become serum biomarkers in several subgroups for the detection of PCA susceptibility.

## Data Availability

All the data generated in the present research is contained in this manuscript.

## Abbreviations

PCA: Prostate cancer
TNF-α: Tumor necrosis factor alpha
ORs: Odds ratios
CIs: Confidence intervals
AUC: Area under curve
MHC III: Major histocompatibility complex
SNPs: Single nucleotide polymorphisms
HWE: Hardy-Weinberg equilibrium
